# The Levels, Speciation, and Influencing Factors of Mercury in MSWI Fly Ashes of a High Geological Background Area

**DOI:** 10.3390/toxics14030226

**Published:** 2026-03-06

**Authors:** Liangliang Yang, Li Tang, Qingfeng Wang, Guangyi Sun, Peng Liu, Tianrong He, Zhonggen Li

**Affiliations:** 1 Key Laboratory of Karst Georesources and Environment, Guizhou University, Ministry of Education, Guiyang 550025, China; gs.liangliangyang23@gzu.edu.cn; 2 State Key Laboratory of Environmental Geochemistry, Institute of Geochemistry, Chinese Academy of Sciences, Guiyang 550081, China; sunguangyi@mail.gyig.ac.cn; 3 Guizhou Research and Designing Institute of Environmental Sciences, Guiyang 550081, China; litang90426@163.com; 4 College of Resources and Environment, Zunyi Normal University, Zunyi 563006, China; qfwang@zync.edu.cn; 5 School of Environmental Studies, China University of Geosciences, Wuhan 430074, China; pengliu@cug.edu.cn

**Keywords:** MSWI-FA, mercury, speciation, distribution patterns, influencing factors

## Abstract

Fly ash (FA) captures most mercury (Hg) in the waste stream during municipal solid waste incineration (MSWI), and the content and speciation of Hg in MSWI fly ash (MSWI-FA) have a significant impact on the ecological environment. In this study, 245 fly ash samples were collected from 18 MSWI facilities in Guizhou Province, a fragile karst area with a high Hg background in Southwest China. The results indicate that total Hg ranged from 0.52 to 444 mg/kg among samples, while the geometric means varied from 0.85 to 223.33 mg/kg for different incinerators, with a weighted geometric mean of 22.14 mg/kg, more than double the national average. Substantial temporal variability in Hg content suggested intermittent inputs of Hg-rich waste into the MSW stream. While positive correlations (*p* < 0.05) were observed between Hg and chlorine, potassium, and cadmium, the moderate to low R^2^ values indicate that Hg enrichment is a complex multi-factor system influenced by heterogeneous waste compositions and transient thermochemical conditions. Speciation analysis revealed that most of the Hg exists in its elemental form (F4), constituting over 80% of the total Hg. However, the water-soluble fraction (F1) and the ion-exchangeable fraction (F2) each reached up to 26–29% in some samples, indicating substantial environmental mobility. These findings demonstrate that MSWI-FA in Guizhou contains elevated Hg levels, posing considerable ecological and environmental risks. Enhancing the classification and management of Hg-containing waste in MSW is critical to mitigating the environmental risks of fly ash, particularly in karst ecological areas.

## 1. Introduction

With ongoing urbanization, the generation of municipal solid waste (MSW) continues to increase. According to the United Nations Environment Programme (UNEP), global urban solid waste production has surpassed 2.1 billion tons per year, of which more than 12% is produced in China [1]. Disposing MSW by incineration can reduce more than 90% of the volume and recover heat, gaining increasing attention among many countries, including China [2,3,4]. Since 2020, incineration has replaced landfill as the primary method for treating household waste in China. By the end of 2024, there were 739 operating municipal solid waste incineration (MSWI) facilities in China with an annual treatment capacity of 219 million tons, accounting for 84.6% of the nation’s total safety disposal [5]. The scale of MSWI in China has become the largest in the world, with China’s installed capacity for MSWI accounting for more than 40% of the world’s total [6]. Every year, China alone produces around 10 million tons of incineration fly ash [7], which contains high levels of heavy metals and dioxin-like persistent organic pollutants, leading to its classification as hazardous waste (HW18, code 772-002-18) under China’s National Catalogue of Hazardous Wastes [8]. Among these contaminants, mercury (Hg) is identified as a priority pollutant under the Minamata Convention on Mercury owing to its high toxicity, strong bioaccumulation potential, and long-range atmospheric transport capability [9], consequently, controlling its emissions from waste incineration facilities has become a critical objective of this international treaty.

Hg in MSW mainly originates from electronic waste (e.g., Hg switches and batteries), fluorescent bulbs, and Hg-laden pharmaceuticals and cosmetics [10,11,12]. An investigation showed that fluorescent lamps and batteries comprise approximately 75% of Hg in urban household waste in China [13], whereas the contribution of fluorescent tubes to Hg input can reach 20.5 tons per year [14]. Under incineration temperatures of 850–1100 °C, nearly all Hg is vaporized and released as elemental mercury (Hg^0^), which subsequently enters the flue gas treatment system [11,15]. In the oxidizing and chloride-rich flue gas environment, Hg^0^ can be oxidized to divalent mercury (Hg^2+^), which may be subsequently adsorbed on fly ash or particulate carbon as particulate-bound mercury [16]. During flue gas treatment, such as semi-dry desulfurization, gaseous Hg^2+^ readily dissolves in lime slurry and is transformed into particulate-bound Hg as the slurry evaporates [15]. Activated carbon injection for dioxin control also captures gaseous Hg, further contributing to particulate-bound Hg formation [17]. Consequently, 60–100% of the Hg in MSW is ultimately captured in fly ash [18,19,20,21]. In addition to total Hg in MSWI fly ash (MSWI-FA), Hg speciation is a crucial factor that should be considered when evaluating its environmental risks [22]. Acid-soluble Hg can be easily leached by rainfall, leading to soil and groundwater contamination, and can potentially enter the food chain. Conversely, reducible Hg can convert to methylmercury (MeHg) under anaerobic conditions, greatly increasing its toxicity [23,24,25]. Therefore, understanding both the concentration and speciation of Hg in MSWI-FA is essential for evaluating its long-term environmental impacts and for developing safe disposal strategies.

Guizhou Province in southwestern China holds a unique position in Hg pollution and geochemical studies due to its abundant Hg mineral resources. This province is situated in the center of the circum-pacific mercuriferous belt, and at least 12 large and super-large Hg mines have been discovered in this province, including Wanshan, Danzhai, Wuchuan, etc. [26]. This province holds 80% (80,000 t of metal Hg) of the nation’s confirmed Hg reserves [26], resulting in soil Hg levels that are significantly higher than those in the majority of other regions in China. For instance, the average concentration of Hg in Guizhou’s soils is 0.151 mg/kg [27], approximately three times higher than the national mean of 0.05 mg/kg [28]. In the Wanshan mining region of Tongren city in eastern Guizhou, average soil Hg levels of 16.94 ± 6.97 mg/kg have been reported [29], with a maximum of 631 mg/kg [30]. Furthermore, Guizhou has a low recovery rate for Hg-containing waste, largely due to inadequate waste sorting and recycling practices. This contributes to substantial variability in Hg levels within MSW. Research conducted by our team in the 2000s indicated that the Hg concentration in the MSW of Guiyang, the provincial capital, could be as high as 46 mg/kg [31]. In addition, karst landscapes cover 61.92% of the province’s land area, making it a highly sensitive ecological system, with widespread soil erosion and rocky desertification. Moreover, the groundwater systems are highly complex; hence, contamination in such areas may trigger severe ecological consequences [32,33].

In recent years, Guizhou Province has experienced a marked increase in building MSWI. By 2020, MSWI accounted for over 50% of waste disposal in this province for the first time, and as of July 2024, a total of 26 incineration facilities had been constructed. By the end of 2024, incineration treated 91.1% of MSW in Guizhou [5]. Nevertheless, studies on Hg in MSWI-FA have largely focused on the more developed coastal areas of China [34,35,36]. A significant knowledge gap remains regarding Hg characteristics in fly ash from regions with high geological Hg backgrounds and optimization of appropriate waste disposal technologies for such areas.

This study focuses on the representative MSWI-FA from Guizhou Province, aiming to elucidate the spatiotemporal distribution of total Hg in fly ash from different localities and to identify the factors influencing its variability. Furthermore, Hg speciation was analyzed through a five-step sequential chemical extraction technique to assess its potential environmental risks, taking into account the unique geological and geomorphological conditions of Guizhou. The findings will provide a scientific basis for the safe disposal of MSWI-FA and offer valuable insights for managing solid waste in other high-Hg regions worldwide.

## 2. Materials and Methods

### 2.1. Sample Collection

**Figure 1:**
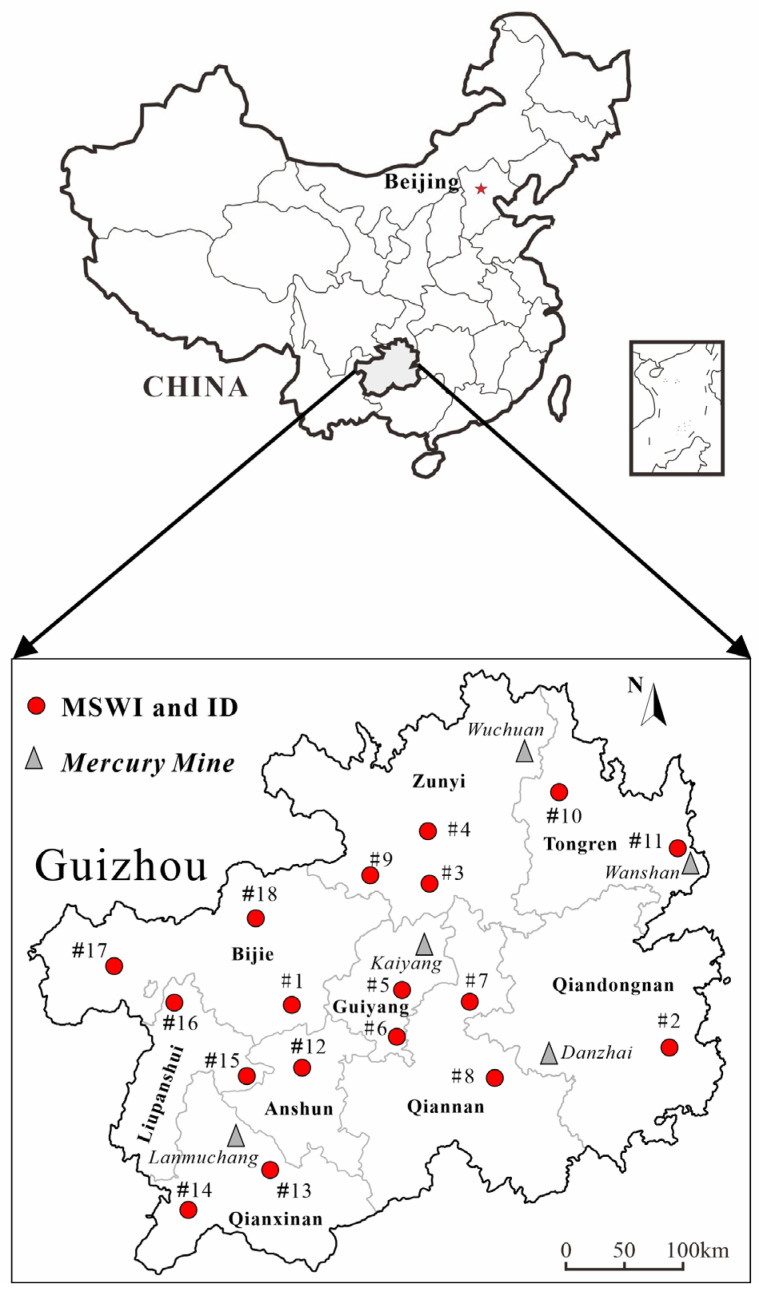
Spatial distribution of the 18 investigated MSWI plants and major mercury mines in Guizhou Province, Southwest China.

Fly ash was collected from 18 MSWI facilities across all nine cities and prefectures in Guizhou Province to ensure comprehensive spatial coverage. The specific geographical locations of these 18 MSWI plants, along with the distribution of major mercury mines in Guizhou, are illustrated in Figure 1.

All facilities employed grate furnace technology and were equipped with comparable flue gas treatment systems. Grate furnace is currently the most important type of incineration for waste disposal in China, accounting for over two-thirds [37], and current household waste incinerators in Guizhou use this technology. The flue gas pollution control facilities adopted the following: selective non-catalytic reduction (SNCR) for NOx removal, semi-dry/dry scrubbing (SDS/DS) for acid gas neutralization, activated carbon injection (ACI) for heavy metal and dioxin capture, and fabric filters (FFs) for particulate collection. The processing capacities of these facilities ranged from 400 to 2400 tons per day (t/d). Key operational information for each facility is summarized in Table 1. Among the 18 plants, facilities #5 and #6 are located in Guiyang, the provincial capital; plants #3, #8, #11, #12, #16, and #18 are located in prefecture-level cities, while plants #1, #2, #4, #7, #9, #10, #13, #14, #15, and #17 are situated in county-level cities. Notably, plant #11, located in eastern Guizhou, is situated approximately 40 km northwest of the Wanshan mercury mine, the largest Hg deposit in China. The Wanshan mine operated for more than two thousand years (from 221 B.C. to 2001) [38]. Currently, there are still many industries related to Hg in the area, such as the production of Hg chemical reagents, the production of Hg catalysts used in PVC production, and the recovery of Hg resources from Hg-containing waste (such as non-ferrous metal smelting sludge, waste mercury catalysts, etc.).

Fly ash was collected from the hopper of baghouse filter of the air pollution control device [39]. Each sample weighed approximately 1 kg. In most facilities, more than five fly ash samples were collected at intervals of 3 to 7 days. In six plants (incinerators#9–#14), 21–49 samples were collected over a period of two to ten months to assess temporal variations in fly ash Hg concentrations. These six facilities were specifically selected for intensive sampling because they represent diverse geographic regions and urban scales within Guizhou, and their consistent operational status during the study period provided a reliable basis for evaluating long-term fluctuations in Hg levels. Overall, 245 fly ash samples were collected between 2022 and 2024.

### 2.2. Total Hg Analysis

The total Hg concentration in the MSWI-FA was determined using inverse aqua regia digestion, followed by cold vapor atomic absorption spectrometry (CVAAS). A 0.1 to 0.5 g portion of fly ash was weighed and digested with 5 mL of inverse aqua regia. This mixture was heated in a 95 °C water bath for 3 h and then diluted to 50 mL using deionized water. After clarification, the supernatant was subsampled and reduced with 0.4 mL of stannous chloride (SnCl_2_, 20% *w*/*w* in 20% HCl). The released Hg^0^ vapor was quantified using a cold vapor atomic absorption spectrometer (F732VJ, Shanghai Huaguang Instrument Co., Ltd., Shanghai, China). The instrument was calibrated daily using a series of mercury standard solutions (0, 0.1, 0.2, 0.5, 1.0, 2.0, and 5.0 ng/mL) prepared from a certified Hg solution reference material (GBW 08617), which is obtained from National Institute of Metrology, Beijing, China. The calibration curves consistently exhibited high linearity, with correlation coefficients (R^2^) exceeding 0.999. A reagent blank and a mid-range calibration standard were analyzed every 10 samples to monitor instrument stability and baseline drift.

Quality control was ensured using certified fly ash reference materials: GSB 07-3662-2019 (116 mg/kg Hg) produced by Institute of Environmental Standard Reference Materials, Environmental Development Center of the Ministry of Ecology and Environment, Beijing, China, and BCR 176R (1.6 mg/kg Hg) provided by Joint Research Centre-Institute for Reference Materials and Measurements of European Commission, Brussels, Belgium. Recoveries ranged from 96.6% to 103.3%, confirming the accuracy of the method. Additionally, 10% of the samples were analyzed in duplicate, yielding relative deviations below 5%, which verified the method’s reproducibility.

### 2.3. Determination of Mercury Speciation in Incineration Fly Ash

Sixteen representative samples from eleven incineration facilities were selected for the Hg species analysis. Samples denoted with suffixes ‘a’ and ‘b’ (e.g., #4a and #4b) represent distinct representative samples collected from the same incineration facility at different sampling intervals to account for potential temporal variability in Hg speciation. The five-step sequential extraction technique proposed by Diao et al. [40] was utilized to identify Hg forms in the fly ash, including water-soluble (F1), ion-exchangeable (F2), acid-soluble (F3), elemental (F4), and sulfide-bound (F5) fractions. A summary of the extraction procedures is provided in Table A1 of the Appendix A.

For the water-soluble fraction, 1 g of fly ash was mixed with 20 mL deionized water and shaken at 120 rpm for 24 h, followed by centrifugation at 4000 rpm for 15 min. The supernatant was filtered to obtain the leachate. The remaining residue was sequentially treated with 20 mL of the extractants listed in Table A1. Each extraction step involved shaking, centrifugation, and filtration under identical conditions to obtain the leachates for steps 2–5. Total Hg in the leachates was measured following U.S. EPA Method 1631. The leachates were acidified to pH < 2 with 3% HCl, oxidized with BrCl, reduced with hydroxylamine hydrochloride and SnCl_2_, pre-concentrated on a gold trap, and analyzed using a cold vapor atomic fluorescence spectrometer (CV-AFS, Brooks Rand Instruments, Seattle, WA, USA).

To evaluate the effectiveness of the five-step sequential extraction process, ΣF1–F5 was compared with total Hg through single-step inverse aqua regia digestion. The average ΣF1–F5-to-THg ratio was 82.69 ± 12.16% (Table A2), indicating acceptable analytical accuracy (refer to the Appendix A for further information).

### 2.4. Determination of Other Parameters

Additional parameters in the fly ash. were also analyzed to identify influencing factors and evaluate chemical characteristics. Specifically, major components (Ca, Mg, K, and Na) and trace metals (Pb, Cd, Zn, Cu, and Mn) were quantified using atomic absorption spectrometry (AAS) and inductively coupled plasma mass spectrometry (ICP-MS), respectively. Metalloids including As, Sb, and Se were determined by atomic fluorescence spectrometry (AFS), while chlorine (Cl) content was measured via ion chromatography (IC). The pH levels were determined using a pH meter at a water-to-solid ratio of 5:1. Detailed descriptions of these analytical methods are provided in the Appendix A.

### 2.5. Data Processing and Statistical Analysis

Data analysis, visualization, and processing were performed using several software tools. Microsoft Excel 365 (Microsoft Corp., Redmond, WA, USA) was utilized for data organization, calculations, and descriptive statistics (such as mean and standard deviation). All figures, including box plots, scatter plots, and correlation diagrams, were generated using OriginPro 2025 (OriginLab Corp., Hampton, MA, USA). Spearman rank correlation analysis was conducted with IBM SPSS Statistics version 29.0.2.0 (IBM Corp., Armonk, NY, USA), with significance levels set at *p* < 0.05, *p* < 0.01, and *p* < 0.001.

## 3. Results and Discussion

### 3.1. THg in Incineration Fly Ash

#### 3.1.1. THg Among Different Incinerators

**Figure 2:**
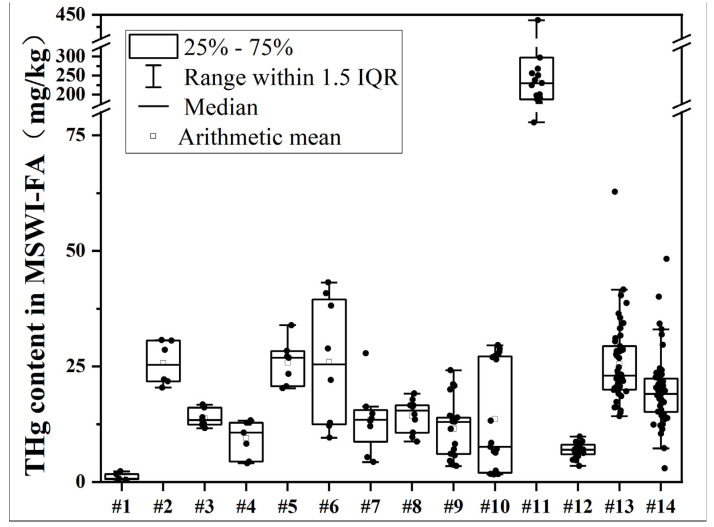
Boxplot of Hg concentrations in fly ash from different incineration plants with more than five samples per facility.

The range of total Hg was from 0.52 mg/kg to 444.38 mg/kg among different samples (Table 2), reflecting a disparity of nearly three orders of magnitude. The geometric mean Hg levels across the different plants varied from 0.85 mg/kg at MSWI #18 to 223.33 mg/kg at MSWI #11 (Figure 2), highlighting significant differences among the MSWIs, and the Hg concentration distribution was found to be right-skewed. Most MSWI plants exhibited relatively low Hg concentrations, generally under 30 mg/kg, but a few, especially MSWI #11, showed exceptionally high levels (Figure 2).

Among the various MSWIs, the fly ash from MSWI #11, situated in northeastern Guizhou, demonstrated a notably high total Hg concentration, averaging at 223.33 mg/kg (refer to Table 2). In the provincial capital of Guizhou, the two facilities—MSWI #5 and MSWI #6—recorded geometric means of 25.44 mg/kg and 22.43 mg/kg, respectively, and MSWI #13 in southwestern Guizhou had a Hg level of 24.32 mg/kg, indicating relatively elevated Hg concentrations. In contrast, the lowest total Hg levels were found in the fly ash from MSWI #1 and MSWI #18 in northwestern Guizhou, with geometric means of 0.98 mg/kg and 0.85 mg/kg, respectively. Since all incineration facilities utilized comparable furnace designs and flue gas management systems, these results suggest that the total Hg levels in fly ash are predominantly determined by the Hg content in the waste being processed, rather than solely by geographic factors, plant capacity, or urban-level (scale) classifications.

Due to variations in the treatment capabilities of different incineration facilities and the highly skewed data, the overall Hg concentration in fly ash from 18 MSWIs was represented using the weighted geometric mean, which was calculated to be 22.14 mg/kg. This figure is marginally above the unweighted geometric mean of 17.16 mg/kg. The discrepancy is largely attributed to certain plants, like MSWI #11, which, despite having a moderate treatment capacity, possess significantly high Hg levels, thus skewing the weighted average. Additionally, major incineration facilities in the provincial capital, Guiyang (MSWI #5 and MSWI #6), also recorded elevated Hg concentrations, contributing to the increased weighted mean. As indicated in Table 3, the weighted geometric mean for Guizhou (22.14 mg/kg) surpasses the national average mercury concentration in MSW-FA, which is reported at 10 mg/kg (with a range of 1–24 mg/kg) across 15 MSWI plants in China [41] and 6.14 mg/kg (with a range of 0.03–85 mg/kg) for a review of 76 plants in China [3]. This suggests that Hg levels in Guizhou’s fly ash are relatively elevated. Similar concentrations have been noted in large cities like Beijing (20.8 mg/kg) and Shenzhen in China (18.6 mg/kg, [42] and South Korea (20.34 mg/kg, [43,44]) (Table 3), while much lower levels were found in a range of cities in China (Zunyi: 2.46 mg/kg, [45]; Chengdu: 2.8 mg/kg, [42]; Hangzhou: 8.43 mg/kg, [46]; Fujian: 5.5 mg/kg, [42]; Chongqing: 5.28 mg/kg, [21]; Shanghai: 13.8–15.8 mg/kg, [47]; Pearl River Delta: 6.67 mg/kg, [48]) and other countries (Japan: 8 mg/kg, [49]; Austria: 12 mg/kg: [50]). Moreover, Hg levels in fly ash from Malmö in Sweden (94 mg/kg, [18]), Switzerland (90–130 mg/kg, [51]), and Shanghai in the 2000s (80 mg/kg) [19] were elevated, potentially due to a higher Hg content (2–4 mg/kg) in MSW during that period. Overall, the Hg content in incineration fly ash from Guizhou Province is considered moderate to high when compared to both domestic and international cases. Notably, MSWI #11 has the highest recorded Hg concentration in incineration fly ash in China to date.

The Hg content in fly ash from incineration is affected by various factors, including the type of incineration technology used, flue gas temperature, air pollution control systems, and the Hg content in the waste being processed [18,20,53,54]. Since all facilities included in this study employed comparable furnace types, air pollution control measures, and sampling locations, the differences observed can be primarily linked to the Hg levels in the waste input. This indicates that there are significant differences in Hg input from household waste between different cities due to factors such as lifestyle habits, usage and recycling levels of Hg containing products, the distribution of Hg-related industries, and soil background values. In Guizhou, items containing Hg, such as fluorescent lamps, Hg thermometers, and button cells, are not always separated from MSW before incineration, resulting in significant fluctuations in Hg concentrations across different cities. Furthermore, the elevated background levels of Hg in soil and solid waste from Hg industries (such as smelting and chemical production) in some cities may add an increased Hg load to daily MSW. For example, street dust in the Wanshan district has been found to contain as much as 30.59 mg/kg of Hg [55], which is collected and sent to incinerator #11 for processing, while other cities in Guizhou typically report street/house dust Hg levels of 0.22–0.58 mg/kg [55,56,57]; in addition, total Hg in mine waste from the Wanshan mining area reached 4.2 to 4400 mg/kg [38,58]. This likely accounts for the notably high Hg concentration in the fly ash from MSWI #11. In Guiyang, the average Hg concentration in MSW was 1.87 mg/kg, with a peak of 46 mg/kg [31], aligning with the elevated Hg levels found in its fly ash. Research on the primary components of Chinese MSW, including food, paper, rubber/plastics, textiles, bamboo, wood, bricks, glass/ceramics, and metals, has revealed that the Hg content in these components is relatively low, ranging from 0.01 to 0.36 mg/kg; however, the Hg concentration in fine mixtures can be significantly higher, reaching levels between 1.07 and 3.72 mg/kg, which constitutes 61.4% to 84.3% of the total Hg input [35]. Research has shown that the average Hg content in household waste in most cities in China is relatively low, ranging from 0.27 to 0.96 mg/kg [59,60], but some cities may have higher levels of waste, reaching 2.5–7.87 mg/kg [19,60]. A case in the United States shows that even in two cities within the same state, the Hg content in urban household waste can differ by up to three times (2.6 ± 0.2 mg/kg versus 0.9 ± 0.2 mg/kg, [52]). Therefore, the Hg content in urban household waste is influenced by multiple factors. By reducing the use of Hg-containing products and the Hg content per unit product, the Hg content in household waste can be significantly reduced. These successful cases have been demonstrated in Western countries [11] and China [13] over the past few decades. For example, the Hg content in China’s domestic waste has shown a continuous declining trend, decreasing from 1.8 mg/kg in 1995 to 0.5 mg/kg in 2015, primarily due to the reduction in Hg levels in batteries [6,13].

#### 3.1.2. Temporal Variation in Total Hg in MSWI-FA

**Figure 3:**
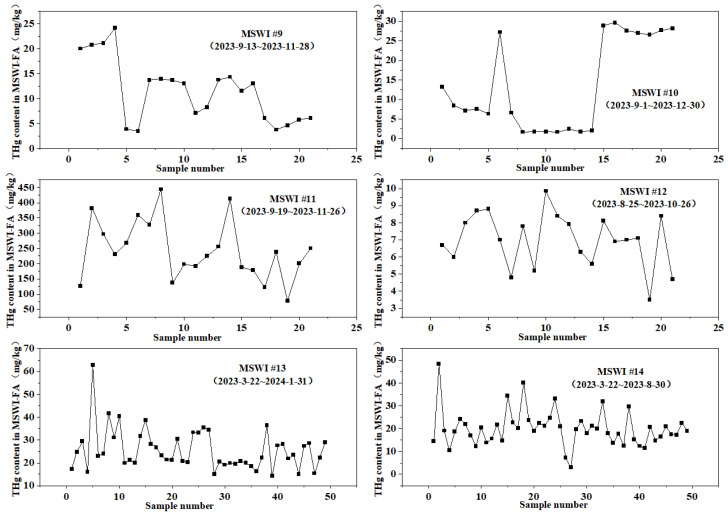
Temporal variation in Hg concentrations in MSWI-FA from the six selected incinerators (#9–#14).

Figure 3 illustrates that the concentrations of Hg in fly ash from six incineration facilities, each with a substantial number of samples (ranging from 21 to 49 per facility), displayed considerable temporal fluctuations. These variations suggest that the Hg levels in fly ash are significantly affected by the changing composition of the waste being processed. The fluctuations observed across the six facilities can be categorized into three distinct types: (1) High-frequency, large-scale fluctuations (MSWI #11), which demonstrated the most pronounced variability among all the plants. Here, total Hg levels could rise from under 100 mg/kg to over 400 mg/kg in a very short period (one week), followed by a swift decrease and subsequent increase. This spike–pulse behavior indicates the sporadic introduction of high-Hg waste materials into the incineration feed, such as used fluorescent bulbs, Hg-laden batteries, broken thermometers, or other local sources rich in Hg (like street dust and various forms of waste [54]). The introduction of such waste batches leads to spikes in Hg levels in the fly ash. (2) Moderate-frequency, moderate-magnitude fluctuations (MSWI #9, #10, #13, #14): These four facilities showed less extreme but still significant variability, with Hg concentrations often varying within a broader range (for instance, MSWI #13 fluctuated between 10 and 100 mg/kg). This pattern highlights the natural diversity found in urban MSW. (3) Relatively stable, low-magnitude fluctuations (MSWI #12), where fluctuations were noted (3.5–9.8 mg/kg) during a two-month period, but both the amplitude and frequency were significantly lower compared to the other five facilities. In summary, while there is temporal variability in Hg levels in fly ash from various cities, the total Hg content in incineration fly ash remains a reliable indicator of the Hg concentrations present in local MSW.

#### 3.1.3. Factors Influencing Total Hg in MSW-FA

**Figure 4:**
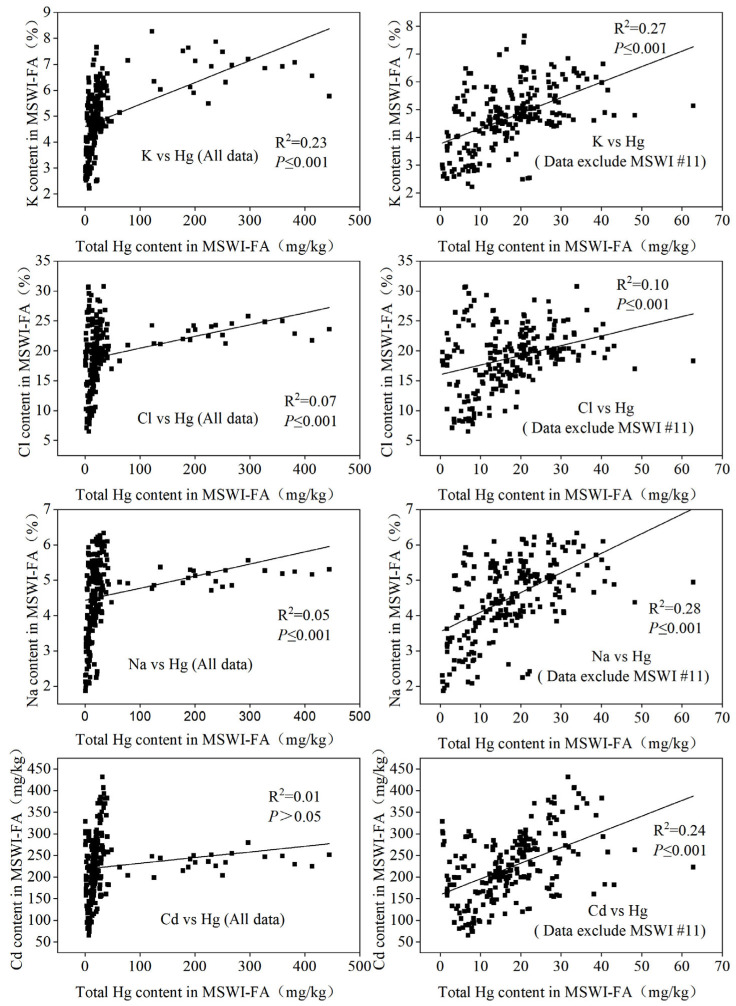
Correlations between Hg and potassium (K), chlorine (Cl), sodium (Na), and cadmium (Cd) in MSW-FA (the (**left**) column shows the data while the (**right**) column shows the data excluding plant #11).

**Figure A1:**
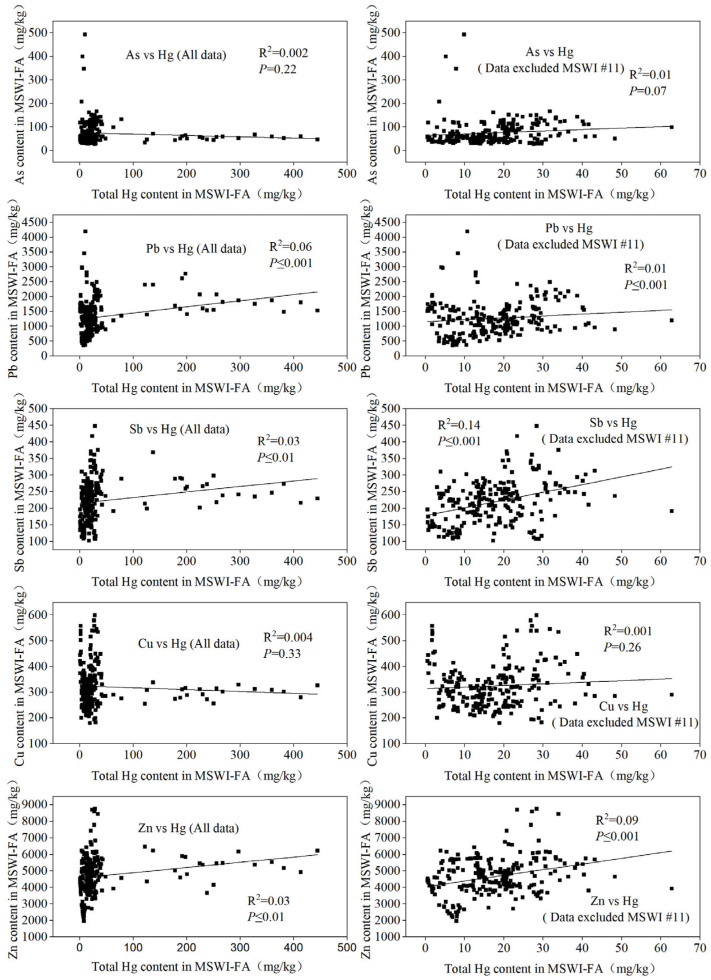

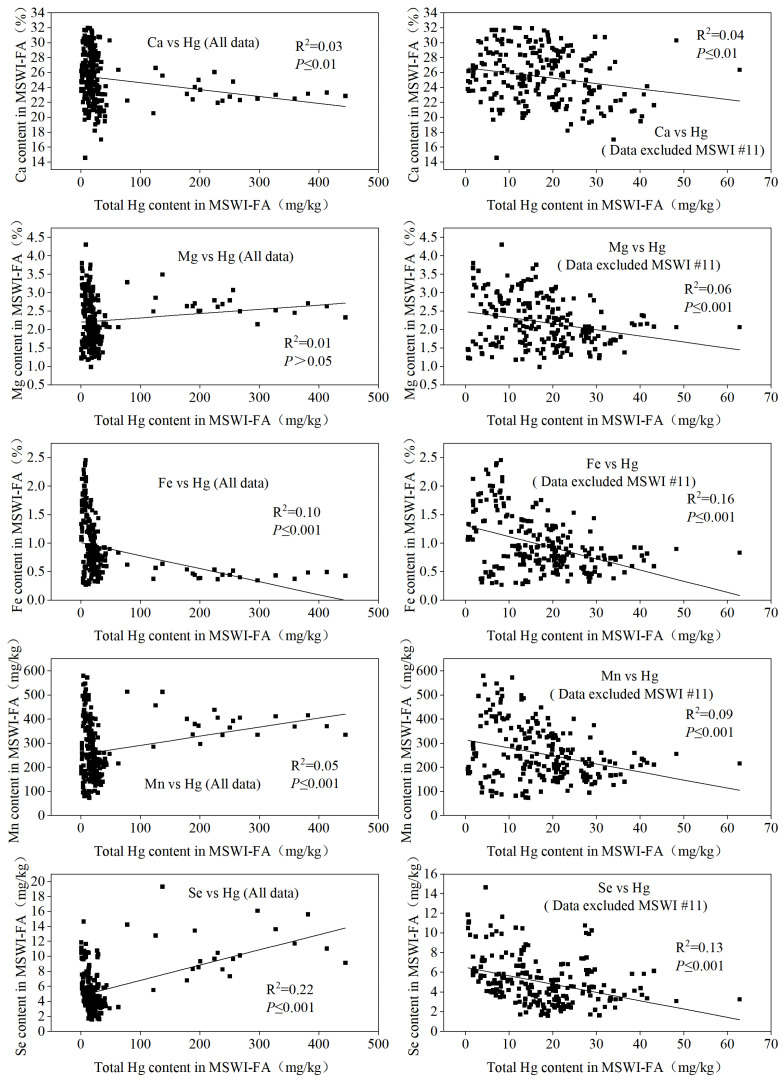
Correlation between Hg and other elements in incineration fly ash.

Figure 4 and Appendix A Figure A1 demonstrate the complex relationships between Hg and various major and trace elements in the fly ashes, highlighting their interactions throughout the incineration process. While some elements show statistically significant correlations, the relatively moderate to low R^2^ values (e.g., R^2^ = 0.10 for Cl and R^2^ = 0.24 for Cd) suggest that Hg enrichment in MSWI-FA is a multi-factor system where no single element dictates its distribution. This variability is primarily driven by the heterogeneous nature of MSW and the diverse physicochemical processes occurring within the flue gas. Excluding data from the high-Hg MSWI #11 facility clarified specific coupling patterns among the remaining 17 plants. Notable positive correlations (*p* < 0.05) were observed between Hg and potassium (K), sodium (Na), chlorine (Cl), and cadmium (Cd). The positive correlation with chlorine (R^2^ = 0.10, *p* < 0.001) is particularly significant; the MSWI-FA in this study had a high average chlorine content of 19.0 ± 4.9% (range 6.4–29.5%), originating from chlorinated plastics and other waste. Elevated chlorine facilitates the formation of gaseous mercury chloride (HgCl_2_) at temperatures below 400 °C [16]. As the flue gas cools, HgCl_2_ tends to condense or adhere to fly ash particles, particularly fine particles that are rich in potassium salts. Consequently, increased levels of Cl and K in fly ash enhance the conversion of gaseous mercury into particulate mercury, leading to its accumulation in the fly ash. Additionally, potassium and sodium exhibited significant positive correlations with Hg; these elements in MSW primarily originate from kitchen waste, especially salt, soy sauce added during the cooking process, and various pickled and processed foods. During incineration, these components (K and Na) largely evaporate and subsequently condense into the fly ash. Chlorine plays a crucial role in the chlorination of Hg, while potassium may serve as a condensation nucleus or indicator of specific components. Sodium, being an alkali metal similar to potassium, may also engage in the adsorption and fixation of Hg. In addition, a strong positive correlation was noted between cadmium and Hg (R^2^ = 0.24, *p* ≤ 0.001), significantly higher than that of lead (Pb, R^2^ = 0.01) and other elements (refer to Appendix A Figure A1), suggesting a close association between Cd and Hg in urban MSW, likely sourced from batteries, electronic waste, and similar materials, undergoing comparable “volatilization–condensation” chemical processes [15]. The correlation coefficients for arsenic (As) and copper (Cu) with Hg were nearly zero, indicating a lack of a significant linear relationship, possibly due to the complex behavior of these elements during incineration, since these elements largely remain in the bottom ash during incineration [61]. Zinc (Zn), antimony (Sb), and lead (Pb) are partially volatile during incineration and exhibit some co-enrichment with Hg, but their correlations are relatively weak (with coefficients ranging from 0.03 to 0.06; see Appendix A Figure A1), likely due to lower volatility or differing sources in daily MSW [19]. Elements showing significant negative correlations with Hg included iron (Fe), calcium (Ca), magnesium (Mg), and manganese (Mn) (see Appendix A Figure A1), which may be introduced through lime slurry used to neutralize acidic gases (e.g., SO_2_ and HCl) from flue gas. In this study, the molar ratios of Ca/S and Ca/Cl_2_ in fly ash from the 18 plants were significantly greater than 1, averaging at 8.55 ± 3.95 (range 3.67–28.52) and 2.64 ± 1.20 (range 0.91–7.81), respectively, indicating an excess of injected lime. As a result, increased lime addition leads to dilution and lower Hg concentrations in fly ash. The pH of the fly ash was also elevated (11.46–11.87, average 11.65 ± 0.08), further confirming the impact of excess lime. Selenium (Se) in fly ash was predominantly negatively correlated with Hg, both of which are volatile at high temperatures, and Se in flue gas may react with gaseous Hg to form mercury selenide [62]. However, in this study, the Se content in fly ash was very low (in most cases less than 5 mg/kg, or even 2 mg/kg), and the enrichment coefficient of selenium was not high compared to the soil selenium background value in Guizhou Province (0.48 mg/kg, [63]). This indicates that there is limited selenium-containing waste mixed into daily waste, and the Hg in incineration fly ash is significantly excessive compared to the soil Hg background value in Guizhou Province (average value of 0.15 mg/kg, [27]). Therefore, the two elements show a negative correlation. Selenium is often found in mercury mines in eastern Guizhou [64]; therefore, if a tiny amount of Hg-containing minerals is mixed into daily household waste, it can lead to an increase in both Hg and Se content, as observed in MSWI #11. Including the unusually high Hg levels from MSWI #11 weakened the correlations between Hg and most elements and revealed outliers. This is mainly due to the exceptionally high Hg input from the feedstock at this facility, obscuring the typical coupling trends between Hg and other elements. This suggests that the Hg content in the incoming waste is the primary factor influencing Hg levels in fly ash. To effectively lower the Hg levels in fly ash, it is essential to enhance the classification of MSW and meticulously separate hazardous wastes containing Hg, thus preventing its entry at the source. China will prohibit the production of Hg-containing thermometers and sphygmomanometers from 1 January 2026. As a result, Hg levels in MSW from this category of Hg-containing materials are expected to decline significantly. However, for waste from high background areas, the sources of Hg still need to be further identified, and separation measures must be implemented to prevent its entry into incineration plants, thereby reducing the amount released into the atmosphere and in incineration fly ash.

### 3.2. Speciation of Mercury in MSW-FA

**Figure 5:**
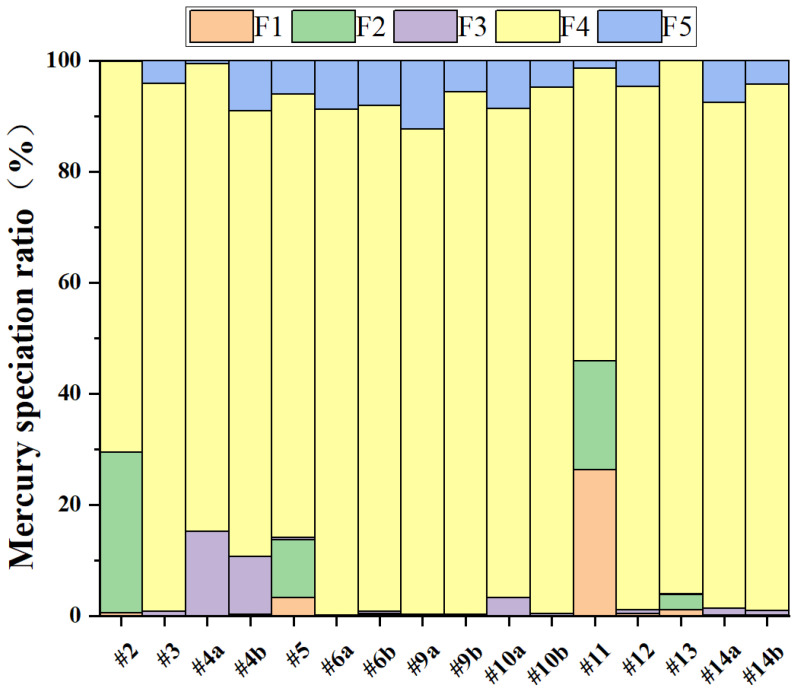
Speciation of Hg in different MSWI-FA samples. (Suffixes ‘a’ and ‘b’ (e.g., #4a, #4b) denote different representative samples collected from the same incineration facility at different sampling intervals).

Figure 5 illustrates a clear and consistent pattern in the distribution of Hg speciation across all sixteen samples, with F4 (elemental mercury) being the most prevalent form, comprising between 52.71% and 95.95% (averaging 86.61%) (Table A3). Out of the 16 samples analyzed (#2 to #14b), only #2, #5, and #11 show F4 proportions below 80%, while the other 13 samples exceed this threshold. Notably, samples #3, #6a, #6b, #9b, #10b, #12, #13, #14a, and #14b report F4 levels surpassing 90–95%. Previous research utilizing sequential selective extraction techniques has similarly found that elemental mercury constitutes the majority of Hg in MSW-FA, representing 65.34–93.73% of the Hg content [47]. Other forms of Hg are present in much smaller amounts. F1 (water-soluble) and F2 (ion-exchangeable) typically make up less than 5% in most samples, with two exceptions: sample #2 has a notably high F2 proportion nearing 30%, while sample #11 shows F1 at approximately 26% and F2 at 20% (Table A3). This indicates that the fly ash from MSWI #11, which contains the highest Hg concentration (over 400 mg/kg), also has the largest shares of water-soluble and ion-exchangeable mercury, totaling 46%. The concentration of water-soluble Hg in the leachate is 5.71 mg/L, significantly exceeding China’s Class V surface water standard (GB 3838-2002 [65], ≤0.001 mg/L) and the integrated wastewater discharge standard in China (GB 8978-1996 [66], ≤0.05 mg/L), underscoring its potential environmental hazard. The fractions of F3 (acid-soluble) and F5 (sulfide-bound) mercury are minimal across all samples, generally remaining below 5% and often undetectable.

Regardless of the total Hg levels in MSW-FA (e.g., #10a, 1.96 mg/kg and #11, 444 mg/kg), F4 remains the dominant mercury species. This suggests that during incineration, the primary stable form of Hg in fly ash is elemental, irrespective of the initial Hg content in the waste. This phenomenon is likely linked to the physicochemical changes that occur as flue gas cools in the presence of excess HCl. While most Hg may convert to Hg_2_Cl_2_ (F4) during incineration and subsequent cooling processing, some samples still retain a notable amount of soluble mercury (F1, potentially as HgCl_2_). Consequently, fly ash from facilities like MSWI #11 not only has elevated total Hg levels but also contains a higher proportion of mobile F1 mercury. Inadequate disposal methods, such as simple landfilling, could result in groundwater and soil contamination, creating significant environmental hazards. Sample #2 (total Hg: 12.32 mg/kg) displays a unique speciation profile, with F2 (ion-exchangeable) representing the largest share among the 16 samples (approximately 30%; Hg concentration in the extract: 0.218 mg/L) and surpassing the integrated wastewater discharge standard (GB 8978-1996 [66], ≤0.05 mg/L) in China more than 4 times, while F4 constitutes about 70%, and other forms are below 1%. The higher proportion of the F2 form of Hg indicates that the Hg adsorbed on fly ash is not firmly bounded and poses a relatively high environmental threat.

## 4. Conclusions

The findings of this study reveal that Hg concentrations in MSWI-FA in Guizhou span three orders of magnitude, with a weighted geometric mean of 22.14 mg/kg across 18 incineration facilities. The highest Hg concentration (over 400 mg/kg) occurred at MSWI #11 in eastern Guizhou, which exceeds the values reported both domestically and internationally, followed by #5 and #6 in the provincial capital. Hg enrichment in fly ash is influenced by chemical reactions during incineration, particularly those involving chlorine and potassium. Sequential extraction results indicate that most Hg in MSWI-FA is present in its elemental fraction (likely as Hg_2_Cl_2_), whereas some samples contain substantial proportions of F1 (water-soluble) and F2 (ion-exchangeable) Hg, which may enhance environmental mobility and associated risks. Therefore, careful management of fly ash disposal is essential to minimize the risk of secondary environmental contamination. Future work will focus on identifying the sources of Hg in MSW, especially high-Hg MSWI-FA. In addition, when disposing of and treating MSWI-FA, such as utilizing it as a raw material for cement production after water-washing, it is advisable to prioritize using fly ash with lower Hg concentrations to reduce secondary Hg emissions.

Despite these observations, it is important to acknowledge that the factors influencing Hg concentrations in MSWI-FA represent a highly complex, multi-variable system. While correlations with elements like Cl, K, Na, and Cd were identified, the relatively low R^2^ values indicate that no single factor provides a definitive explanation for the observed Hg variability. This suggests that the interplay between the heterogeneous composition of MSW-driven by localized waste sources and the presence of high geological Hg backgrounds-and the transient thermochemical conditions during incineration predominates over simplified linear relationships. Consequently, future investigations should transition from simple correlation analyses to multi-factor modeling and source-tracing studies to provide more conclusive insights into the mechanisms governing Hg enrichment in high-background regions.

## Figures and Tables

**Table 1:** Basic information of the 18 grate furnace MSWI plants investigated.

ID of Incineration Plant	Location in Guizhou and Urban Level	Installed Capacity (t/d)
MSWI #1	Northwestern, county level	600
MSWI #2	Southeastern, county level	700
MSWI #3	Northern, city level	1500
MSWI #4	Northern, county level	400
MSWI #5	Central, provincial capital	2250
MSWI #6	Central, provincial capita	2400
MSWI #7	Southern, county level	600
MSWI #8	Southern, city level	600
MSWI #9	Northern, county level	1200
MSWI #10	Northeastern, county level	500
MSWI #11	Northeastern, city level	600
MSWI #12	Central-west, city level	1100
MSWI #13	Southwestern, county level	600
MSWI #14	Southwestern, county level	1200
MSWI #15	Western, county level	500
MSWI #16	Western, city level	1200
MSWI #17	Northwestern, county level	800
MSWI #18	Northwestern, city level	1000

**Table 2:** Statistics of total Hg concentrations (mg/kg) in MSW-FA in this study.

ID of Incinerators	Number of Samples	Minimum	Maximum	Geometric Mean	Standard Deviation
MSWI #1	5	0.52	2.33	0.98	0.8
MSWI #2	6	20.43	30.7	25.35	4.75
MSWI #3	7	11.63	16.73	13.72	1.92
MSWI #4	7	4.07	13.29	8.59	3.97
MSWI #5	7	20.29	33.93	25.44	4.79
MSWI #6	8	9.59	43.17	22.43	13.74
MSWI #7	8	4.32	27.86	11.66	7.25
MSWI #8	10	8.74	19.13	13.92	3.59
MSWI #9	21	3.47	24.16	9.77	6.32
MSWI #10	21	1.65	29.64	7.88	11.83
MSWI #11	21	77.77	444.38	223.33	98.58
MSWI #12	21	3.53	9.85	6.8	1.6
MSWI #13	49	14.26	62.79	24.32	8.85
MSWI #14	49	2.97	48.29	18.43	7.85
MSWI #15	1	3.43	3.43	3.43	--
MSWI #16	1	16.96	16.96	16.96	--
MSWI #17	1	9.25	9.25	9.25	--
MSWI #18	2	0.69	1.05	0.85	0.26
Total	245	0.52	444.38	17.16	69.88

**Table 3:** Comparison of total Hg in MSWI-FA and MSW across different regions worldwide.

Regions	Range of Hg in MSW-FA (mg/kg)	Average Hg in MSW-FA (mg/kg)	Hg in MSW (mg/kg)	References
18 MSWIs in Guizhou, China	0.52–444.38	22.14 a	--	This study
15 MSWIs in China	1–24	10	--	[41]
76 data in China	0.03–84.6	6.14 b	--	[3]
Beijing, China	--	20.8 ± 0.7	--	[42]
Chengdu, China	--	2.8 ± 0.2	--	[42]
Fujian, China	--	5.5 ± 0.2	--	[42]
2 MSWIs in Shanghai, China	3.8–44.8	13.8; 15.8	--	[47]
2 MSWIs in Shanghai, China	20–160	80	0–2.5	[19]
Shenzhen, China	--	18.6 ± 0.6	--	[42]
Zunyi, China	2.06–3.17	2.46	--	[45]
Hangzhou, China	--	8.43	--	[46]
Chongqing, China	--	5.28	0.37	[21]
7 MSWIs in Guangdong, China	0.89–13.71	6.67	0.066–0.636	[48]
South Korea	--	20.34	0.42	[43,44]
19 MSWIs in Japan	0.03–41	8	--	[49]
Sweden	--	94	2	[18]
Austria	--	12 ± 3	--	[50]
Switzerland	90–130	--	2.9–4.2	[51]
Two counties in United States	--	--	0.9–2.6	[52]

a, weighted geometric mean; b, geometric mean; -- means no data available.

## Data Availability

The raw data supporting the conclusions of this article will be made available by the authors on request.

## Appendix A. Methods for Determining Other Components and Parameters in Fly Ash

### Appendix A.1. Determination of Arsenic (As), Antimony (Sb), and Selenium (Se)

Following the procedure outlined in [67], 0.5 g of municipal solid waste incineration fly ash (MSWI-FA) was weighed and transferred into a 50 mL glass colorimetric tube. Subsequently, 10 mL of aqua regia (in a 1:1 ratio with deionized water) was added. The tube was then placed in a water bath at 95 °C for 2 h, with intermittent shaking during the process. Once cooled, the volume was adjusted to 50 mL with ultrapure water, ensuring thorough mixing. The supernatant was collected for further analysis. To determine arsenic (As) and antimony (Sb), a specific volume (for instance, 5 mL) of the supernatant was transferred into a 10 mL plastic volumetric flask. Subsequently, 1 mL of hydrochloric acid (HCl) and 1 mL of thiourea solution were added, and the flask was filled to the mark with ultrapure water and mixed thoroughly. The mixture was allowed to sit at room temperature for 30 min before measuring the concentrations of As and Sb using a Haiguang AFS-9530 atomic fluorescence spectrometer, after reduction with potassium borohydride. For selenium (Se) analysis, the supernatant obtained from the aqua regia digestion could be directly analyzed using the AFS-9530 without the need for HCl and thiourea treatment.

### Appendix A.2. Determination of Chlorine

Following the method outlined in [68], 100 mg of fly ash was weighed and placed into a crucible; we then covered the sample with 3 g of an Eschka mixture, which was prepared by thoroughly mixing magnesium oxide (MgO) and anhydrous sodium carbonate (Na_2_CO_3_) in a 2:1 mass ratio. The crucible was then placed in a muffle furnace and heated to 680 °C for a duration of 3 h. After cooling to ambient temperature, the ignited material from the crucible was transferred into a beaker with hot deionized water. The residue in the beaker was filtered using qualitative filter paper. The filtered solution was adjusted to a final volume of 250 mL with deionized water. From this solution, 3 mL was taken, diluted five times with ultrapure water, and analyzed using ion chromatography under specified conditions: the eluent consisted of sodium carbonate at 324 mg/L and sodium bicarbonate at 64 mg/L, with a suppressor solution of 1% H_2_SO_4_ (10 mL/L). The measurement was conducted using a Metrohm 863 Compact Autosampler ion chromatograph.

### Appendix A.3. Determination of Other Metal Elements

The digestion method was adapted from [69]. A total of 50 mg of the MSWI-FA sample was placed into a PTFE container, followed by the addition of 1 mL of HF and 1 mL of HNO_3_. The container was then sealed and positioned within a stainless-steel bomb for digestion in an oven set at 190 °C for over 24 h. After cooling, the container was taken out, and the solutions were evaporated on a hotplate at 180 °C. Subsequently, 0.5 mL of HNO_3_ was introduced, and evaporation continued until the mixture was completely dry. Following the drying process, 500 ng of a Rh internal standard solution, along with 2 mL of HNO_3_ and deionized water, was added to the container. The container was returned to the steel bomb and heated at 140 °C for 5 h. After cooling, the solution was mixed thoroughly, and 0.4 mL was transferred to a centrifuge tube, which was then diluted to a final volume of 10 mL. The resulting digested solution was analyzed for Pb, Cd, Zn, Cu, and Mn using inductively coupled plasma mass spectrometry (ICP-MS, Analytik Jena, Jena, Germany), while Ca, Mg, K, and Na were quantified using an atomic absorption spectrometer (AA-6880, Shimadzu Corporation, Kyoto, Japan).

### Appendix A.4. pH Determination of Incineration Fly Ash

At ambient temperature, MSWI-FA samples were mixed with deionized water in a mass ratio of 5:1 (for example, 6 g of fly ash to 30 mL of water) and shaken for 30 min. The mixture was then allowed to settle for 2 h before the pH of the supernatant was measured using a pH meter (SX836, Shanghai Sanxin Instrumentation Inc., Shanghai, China).

**Table A1:** Analysis of different Hg species in MSWI-FA using a five-step sequential extraction method [40].

Step	Extractant	Experimental Conditions	Extracted Mercury Species	Possible Mercury Compounds
1	Deionized water	Room temperature, 24 h	F1, water-soluble fraction	HgCl_2_, HgSO_4_
2	0.5 M NH_4_Cl	Room temperature, 20 h	F2, ion-exchangeable fraction	Adsorbed Hg^2+^
3	0.5 M HCl	Room temperature, 12 h	F3, acid-soluble fraction	HgO, HgSO_4_
4	50% (*v*/*v*) HNO_3_	Room temperature, 20 h	F4, elemental fraction	Hg^0^, Hg_2_Cl_2_
5	Aqua regia	60 °C, 12 h	F5, sulfide-bound fraction	HgS, m·HgS

**Table A2:** Speciation of Hg in selected MSWI-FA samples.

Sample ID	F1 mg/kg	F2 mg/kg	F3 mg/kg	F4 mg/kg	F5 mg/kg	ΣF1–F5 mg/kg	Total Hg mg/kg	ΣF1–F5/Total Hg (%)
#2 *	0.08	4.23	0.00	10.29	0.02	14.62	20.43	71.56
#3	0.00	0.00	0.09	10.07	0.44	10.60	12.67	83.69
#4a	0.00	0.00	0.55	3.05	0.02	3.62	4.39	82.50
#4b	0.01	0.01	1.11	8.57	0.96	10.66	13.32	80.04
#5	0.73	2.38	0.08	18.08	1.35	22.62	28.75	78.67
#6a	0.00	0.00	0.00	5.70	0.55	6.26	9.59	65.27
#6b	0.04	0.09	0.09	26.38	2.35	28.95	38.36	75.48
#9a	0.01	0.00	0.00	2.72	0.39	3.11	4.59	67.75
#9b	0.00	0.00	0.02	12.49	0.74	13.25	13.82	95.89
#10a	0.00	0.00	0.07	1.82	0.18	2.07	1.84	112.86
#10b	0.00	0.00	0.08	22.47	1.14	23.69	28.61	82.81
#11	113.87	85.04	0.03	228.31	5.86	433.11	441.19	98.17
#12	0.03	0.00	0.05	7.58	0.37	8.04	8.93	90.07
#13	0.33	0.79	0.07	28.42	0.01	29.62	40.93	72.37
#14a	0.00	0.00	0.03	2.32	0.19	2.55	3.00	84.72
#14b	0.00	0.01	0.18	18.11	0.80	19.10	23.54	81.13

Note: * indicates that the sample is from MSWI #2; suffixes ‘a’ and ‘b’ (e.g., #4a, #4b) denote different representative samples collected from the same incineration facility at different sampling intervals.

**Table A3:** Percentage (%) of each mercury speciation form (F1–F5) relative to total Hg in MSWI-FA samples.

Sample ID	F1	F2	F3	F4	F5
#2 *	0.55	**28.93**	0.00	70.38	0.14
#3	0.00	0.00	0.85	95.00	4.15
#4a	0.00	0.00	**15.19**	84.25	0.55
#4b	0.09	0.09	**10.41**	80.39	9.01
#5	3.23	**10.52**	0.35	79.93	5.97
#6a	0.03	0.03	0.06	91.18	8.71
#6b	0.14	0.31	0.31	91.12	8.12
#9a	0.16	0.00	0.05	87.40	12.38
#9b	0.00	0.03	0.16	94.26	5.55
#10a	0.00	0.00	3.30	88.03	8.67
#10b	0.00	0.00	0.34	94.85	4.81
#11	**26.29**	**19.63**	0.01	52.71	1.35
#12	0.38	0.05	0.66	94.32	4.60
#13	1.11	2.67	0.24	95.95	0.03
#14a	0.00	0.07	1.36	91.10	7.47
#14b	0.00	0.05	0.94	94.82	4.19
Minimum	0.00	0.00	0.00	52.71	0.03
Maximum	26.29	28.93	15.19	95.95	12.38
Arithmetic mean	2.00	3.90	2.14	86.61	5.36
Standard deviation	6.32	8.28	4.19	11.11	3.50

Note: * indicates that the sample is from MSWI #2; suffixes ‘a’ and ‘b’ denote different representative samples collected from the same facility at different times to reflect temporal variations. The data highlighted in bold indicate obviously high proportions of F1, F2, or F3 forms.
